# Clade Ia Monkeypox Virus Linked to Sexual Transmission, Democratic Republic of the Congo, August 2024

**DOI:** 10.3201/eid3105.241690

**Published:** 2025-05

**Authors:** Jean-Claude Makangara-Cigolo, Kelly-Michel Kenye, Lygie Lunyanga, Daan Jansen, Eddy Kinganda-Lusamaki, Sifa Kavira, Adrienne Amuri-Aziza, Emile Malembi, Yvon Anta, Prince Akil-Bandali, Emmanuel Lokilo-Lofiko, Emmanuel Hasivirwe Vakaniaki, Sabin Sabiti Nundu, Elisabeth Pukuta-Simbu, Princesse Paku-Tshambu, Rilia Ola-Mpumbe, Raphael Lumembe-Numbi, Gabriel Kabamba-Lungenyi, Gradi Luakanda, Cris Kacita, Robert Shongo-Lushima, Dieudonné Mwamba, Áine O’Toole, Sydney Merritt, Megan Halbrook, Daniel Mukadi-Bamuleka, Nicole A. Hoff, Nicola Low, Isaac Bogoch, Muge Cevik, Gregg Gonsalves, Souradet Shaw, Lorenzo Subissi, Laurens Liesenborghs, Ahidjo Ayouba, Martine Peeters, Eric Delaporte, Sofonias Tessema, Anne W. Rimoin, Jean-Jacques Muyembe-Tamfum, Andrew Rambaut, Koen Vercauteren, Steve Ahuka-Mundeke, Jason Kindrachuk, Tony Wawina-Bokalanga, Placide Mbala-Kingebeni

**Affiliations:** Institut National de Recherche Biomédicale, Kinshasa, Democratic Republic of the Congo (J.-C. Makangara-Cigolo, K.-M. Kenye, L. Lunyanga, E. Kinganda-Lusamaki, S. Kavira, A. Amuri-Aziza, Y. Anta, P. Akil-Bandali, E. Lokilo-Lofiko, E. Hasivirwe Vakaniaki, S. Sabiti Nundu, E. Pukuta-Simbu, P. Paku-Tshambu, R. Ola-Mpumbe, R. Lumembe-Numbi, G. Kabamba-Lungenyi, G. Luakanda, D. Mukadi-Bamuleka, J.-J. Muyembe-Tamfum, S. Ahuka-Mundeke, T. Wawina-Bokalanga, P. Mbala-Kingebeni); Cliniques Universitaires de Kinshasa, Université de Kinshasa, Kinshasa (J.-C. Makangara-Cigolo, K.-M. Kenye, E. Kinganda-Lusamaki, R. Lumembe-Numbi, G. Kabamba-Lungenyi, D. Mukadi-Bamuleka, J.-J. Muyembe-Tamfum, S. Ahuka-Mundeke, T. Wawina-Bokalanga, P. Mbala-Kingebeni); University of Bern, Bern, Switzerland (J.-C. Makangara-Cigolo, N. Low); Institute of Tropical Medicine, Antwerp, Belgium (D. Jansen, E. Hasivirwe Vakaniaki, L. Liesenborghs, K. Vercauteren, T. Wawina-Bokalanga); TransVIHMI, Université de Montpellier, INSERM, IRD, Montpellier, France (E. Kinganda-Lusamaki, A. Ayouba, M. Peeters, E. Delaporte); Hemorrhagic Fever and Monkeypox Program, Ministry of Health, Kinshasa (E. Malembi, R. Shongo-Lushima); Institut National de Santé Publique (INSP), Kinshasa (C. Kacita, D. Mwamba); Institute of Ecology and Evolution, University of Edinburgh, Edinburgh, Scotland, UK (A. O’Toole, A. Rambaut); Jonathan and Karin Fielding School of Public Health, University of California, Los Angeles, CA, USA (S. Merritt, M. Halbrook, N.A. Hoff, A.W. Rimoin); Toronto General Hospital, University Health Network, Toronto, Ontario, Canada (I. Bogoch); University of St. Andrews, St. Andrews, Scotland, UK (M. Cevik); Yale School of Public Health, New Haven, Connecticut, USA (G. Gonsalves); University of Manitoba, Winnipeg, Manitoba, Canada (S. Shaw, J. Kindrachuk); World Health Organization, Geneva, Switzerland (L. Subissi); KU Leuven, Leuven, Belgium (L. Liesenborghs); Africa Centers for Disease Control and Prevention, Addis Ababa, Ethiopia (S. Tessema)

**Keywords:** mpox, monkeypox virus, MPXV, sexually transmitted infections, zoonoses, viruses, clade I, fatal, case investigation, genome sequencing, DRC, Democratic Republic of the Congo

## Abstract

Several concurrent mpox outbreaks are ongoing in the Democratic Republic of the Congo. We report a case of severe clade Ia mpox in an adult woman with indeterminate HIV status who died 16 days after symptom onset. She self-identified as a sex worker and had spent time in the capital city, Kinshasa.

Mpox, a zoonotic viral disease caused by monkeypox virus (MPXV), is endemic in forested regions of central and western Africa. In recent years, disease burden has increased notably in mpox-endemic areas, alongside rapid geographic spread to nonendemic regions worldwide (*1*). 

Historically, clade I mpox outbreaks have been predominantly driven by zoonotic transmission (*2*). In 2024, we reported a cluster of clade I mpox cases associated with sexual contact in Kwango Province, Democratic Republic of the Congo (DRC) (*3*). Although this outbreak subsided spontaneously, the emergence of clade Ib in eastern DRC has been associated with sustained human-to-human transmission (*4*,*5*). In parallel, clade Ia MPXV, associated primarily with zoonotic transmission, has been responsible for most mpox cases in DRC during the current public health emergency and has spread rapidly across the country.

Genomic investigations of mpox cases in 2024 found clades Ia and Ib in Kinshasa Province, a large urban center with international connectivity (*6*). This situation raises concerns about the potential public health effects on zoonotic and nonzoonotic transmission of clade Ia. In this study, we describe a fatal case of mpox caused by clade Ia, suspected to have been acquired through sexual contact, in a woman returning to Kwango from Kinshasa. Samples were collected under routine mpox surveillance, exempt from ethical approval. The Ethics Committee of Kinshasa School of Public Health (ESP-UNIKIN, nos. ESP/CE/05/2023 and ESP/CE/47/2023) and the Health Research Ethics Board at the University of Manitoba (HS25837) approved data use for this publication.

## The Study

A woman (>20 years of age) with no noteworthy known medical history was transferred from a local health center to a reference hospital because of generalized cutaneous pustule-like lesions that appeared in mid-2024. She had no history of smallpox vaccination. The patient self-identified as a sex worker and had recently returned from a 6-month stay in Kinshasa, where she had multiple casual sexual partners. She reported no recent contact with persons with confirmed or suspected mpox and no recent contact with wildlife. Her first symptom was fever, followed by vulval rash and itching, which began in Kinshasa. Acetaminophen, amoxicillin, and clotrimazole vaginal pessaries were given, but no diagnostic tests were performed. Observing no improvement 4 days after symptoms started, she returned home from Kinshasa by bus. She consulted a local health center 2 days later for fever, joint pain, and generalized cutaneous and genital lesions. She was admitted and treated with ceftriaxone, vitamin C, and cetirizine before transfer 3 days later by motorcycle to the reference hospital.

At admission, clinical examination revealed fever, joint tenderness, and generalized and genital pustular lesions. Suspecting mpox, the physician placed the patient in isolation. HIV rapid diagnostic test results in 2 different health facilities were discordant. The first test at the health center was negative using the Determine HIV-1/2 kit (Abbott, https://www.abbott.com). The second test, performed 3 days later at the hospital using Uni-Gold kit (Trinity Biotech, https://www.trintybiotech.com), returned a positive result. A PCR test for HIV was not performed (Table). She was treated with artemisinin-based combination therapy for malaria and antibiotic drugs (ceftriaxone followed by lincocin) and topical antiseptic solutions to prevent secondary bacterial infections arising from skin lesions. She also received oxygen therapy for signs of respiratory distress, acetaminophen for fever, and intravenous fluids for dehydration. Despite those interventions, the patient died 16 days after symptom onset.

**Table:** Laboratory results for patient with clade Ia monkeypox virus linked to sexual transmission, Democratic Republic of the Congo, August 2024

Laboratory test	Result (reference range)	Interpretation
Peripheral blood smear	1 to 10 trophozoites in 100 microscopic fields	Positive for malaria
Hemoglobin level	12 g/dL (10.5–13.5 g/dL)	Normal
Leukocytes	17,000 cells/µL (4,000–12,000 cells/µL)	Abnormal
Erythrocyte sedimentation	30 mm/h (<20 mm/h)	Abnormal

A case investigation team from INRB (Institut National de Recherche Biomédicale) was mobilized during a monitoring and surveillance visit on the day the patient died. The patient was in respiratory distress and had lesions at varying stages of development, some with crusting, leading to a diagnosis of mpox complicated by acute respiratory distress syndrome. Counting lesions manually was not possible because of the generalized distribution and high number. Crust and vesicle swab samples were collected and sent to INRB for diagnosis. Mpox was confirmed by PCR using pan-orthopoxvirus and MPXV-generic primers (*7*,*8*). PCR results indicated amplification cycle threshold values of 16.08 in crust samples and 15.89 in vesicle samples for the pan-orthopoxvirus primers and cycle threshold values of 14.45 in crust samples and 15.80 in vesicle samples for the MPXV-generic primers (using the same samples). 

After PCR confirmation, we performed whole-viral genome sequencing using the clade IIb tiling sequencing protocol (https://www.protocols.io/view/monkeypox-virus-multiplexed-pcr-amplicon-sequencin-5qpvob1nbl4o/v2) and prepared the library on the basis of the Illumina DNA Prep protocol. We loaded the final enriched libraries onto an Illumina iSeq100 (https:/www.illumina.com). We generated MPXV consensus genomes by processing FASTQ files using CZid pipeline (https://czid.org); we used clade I MPXV genome (GenBank accession no. NC_003310.1) as reference. We used the Nextclade online tool (https://clades.nextstrain.org) to assign the clade of MPXV genomes. In addition, we used SQUIRREL to align sequences (https://github.com/aineniamh/squirrel) and inferred a maximum-likelihood phylogeny using IQ-TREE version 2.1.4 (https://github.com/Cibiv/IQ-TREE) with the Kimura 3-substitition plus empirical base frequencies plus invariant sites model as the best fit. Branch support was estimated by the ultrafast bootstrap approximation with 10,000 replicates (*9*). The phylogenetic tree showed that both sequences from the patient were closely related and cluster with clade Ia MPXV sequences from the ongoing outbreak in Kinshasa (Figure). This finding is consistent with severe mpox caused by clade Ia MPXV acquired during the patient’s stay in Kinshasa.

**Figure:**
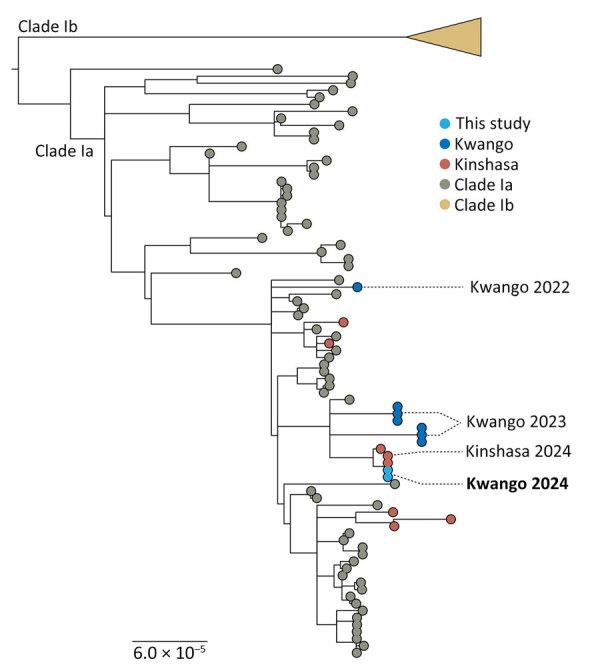
Phylogenetic tree of mpox virus sequences from patient with clade Ia mpox virus linked to sexual transmission and reference sequences, Democratic Republic of the Congo, August 2024.

As part of routine contact tracing, 37 contacts were identified: 10 family members, 4 healthcare workers, 20 friends, and 3 sexual partners; 19 were high-risk contacts. No clinical symptoms of mpox were identified among contacts after 21 days of follow-up. 

## Conclusions

This case report describes fatal mpox caused by clade Ia MPXV in a woman with indeterminate HIV test results and early onset of genital symptoms. This finding could represent HIV seroconversion syndrome in the context of mpox infection, given the history of sexual contacts, clinical symptoms, location of lesions, and discordant HIV test results. In this resource-limited setting, details from clinical examination and laboratory investigations were insufficient to exclude other comorbidities. A false-positive HIV test result is also possible, given the low prevalence of HIV in the general population (15–49 years of age) in the DRC (0.5%–0.7%) (*10*). HIV prevalence of 7.5% was estimated among the key population of sex workers in 2023 (*10*). Given that the sex worker population might be ≈1% in DRC (*11*), this group might be at risk for poor outcomes from mpox, especially if HIV testing rates are low (*12*). PCR testing confirmed the mpox diagnosis, and whole-viral genome sequencing identified clade Ia MPXV. 

This report highlights the potential severity of mpox in DRC and the need to mobilize the community to mitigate spread. The patient was a sex worker and had lived in Kinshasa, from where we reported co-circulation of clades Ia and Ib MPXV during July–August 2024 (*6*). This fact underscores increasing concerns about the expansion of MPXV in DRC, including to a large urban center with extensive regional and international connections, and the changing epidemiology for clade I MPXV. Although MPXV transmission through sexual contact has been predominantly associated with clade Ib (*4*), this case of clade Ia MPXV infection might have been linked to sexual contact, as evidenced by presence of genital lesions. The findings are consistent with our previous report of a cluster of mpox cases associated with sexual contacts in Kwango Province, which also involved clade Ia (*3*).

In summary, this report highlights the importance of prompt diagnosis and public health intervention, specifically among high-risk groups, to prevent the spread of mpox, as well as early diagnosis and management of HIV infection. In regions where mpox is emerging, healthcare providers must maintain a high index of suspicion, particularly in patients with vesiculopustular rash and systemic symptoms.
